# Obesity and tobacco smoking are independently associated with poor patient-reported outcomes in SLE: a cross-sectional study

**DOI:** 10.1007/s00296-024-05546-z

**Published:** 2024-03-07

**Authors:** Alvaro Gomez, Ioannis Parodis, Christopher Sjöwall

**Affiliations:** 1 Division of Rheumatology, Department of Medicine Solna, Karolinska Institutet, Stockholm, Sweden; 2 Department of Gastroenterology, Dermatology, and Rheumatology, Karolinska University Hospital, Stockholm, Sweden; 3 Department of Rheumatology, Faculty of Medicine and Health, Örebro University, Örebro, Sweden; 4 Department of Biomedical and Clinical Sciences, Division of Inflammation and Infection/Rheumatology, Linköping University, Linköping, Sweden

**Keywords:** Systemic lupus erythematosus, Obesity, Smoking, Patient reported outcome measure, Fatigue, Pain

## Abstract

We investigated associations of obesity and tobacco smoking with health-related quality of life (HRQoL), pain, fatigue, and functional impairment in systemic lupus erythematosus (SLE). Furthermore, we explored whether there was an effect modification between these two factors. We included adult SLE patients from the Linköping University Hospital (*n* = 325) in the present cross-sectional analysis. We further included population-based controls and performed cardinality matching to balance age and sex distributions with cases (*n* = 224). HRQoL was assessed with the EQ-5D index score; pain, fatigue, and overall SLE-related health state with visual analogue scales (VAS; 0 [best] to 100 [worst]); and functional impairment with the HAQ-DI. Unacceptable outcomes were defined as VAS scores corresponding to the 90th percentile derived from the matched controls. SLE patients reported worse scores than controls in all measures, and approximately 30% experienced unacceptable outcomes. When compared with normal-weight, obese SLE patients reported lower HRQoL, and greater functional impairment and risk of unacceptable pain (OR: 3.2; 95% CI 1.6–6.7) and fatigue (OR: 2.1; 95% CI 1.0–4.3). Similarly, the current smokers reported higher levels of functional impairment and a greater risk of unacceptable pain (OR: 3.8; 95% CI 1.8–8.2) and fatigue (OR: 2.8; 95% CI 1.3–5.9) than never smokers. The associations were independent of age, sex, disease duration, disease activity, and organ damage. There was no evidence of a synergistic effect between increased BMI and smoking on any outcome. In summary, obesity and smoking are risk factors for unacceptable patient-reported outcomes in SLE, regardless of clinical activity.

**Supplementary Information:**

The online version contains supplementary material available at 10.1007/s00296-024-05546-z.

## Introduction

Systemic lupus erythematosus (SLE) is a systemic autoimmune disease that can affect most organ systems with a varying degree of severity and exhibits unpredictable disease course patterns. The heterogeneity and the complexity of SLE impose challenges when monitoring patients over time, with discrepancies in disease evaluation reported by healthcare providers and SLE patients being common [1, 2]. Therefore, a comprehensive understanding of a patient’s health state requires the measurement of patient-reported outcomes (PROs), to capture highly relevant disease facets which otherwise may be omitted [3].

Even though several drugs used in the management of SLE decrease disease activity, prevent damage progression [4–6], and improve patient-reported health-related quality of life (HRQoL) [7–10], a substantial proportion of patients still fail to achieve an optimal health state as per self-reports. Among patients who achieve clinical response to treatment in randomised clinical trial (RCT) settings, up to one third report adverse physical HRQoL outcomes, and one fourth report severe fatigue [11]. In this scenario, non-pharmacological interventions constitute appealing strategies for improving PROs in these patients [12].

Obesity and tobacco smoking are modifiable factors that could be targeted to improve PROs. Obesity has been associated with impaired HRQoL, pain, and fatigue [13–16]. Using data from two international RCTs, we previously reported associations between obesity and clinically important impairments in physical aspects of HRQoL, fatigue, and social functioning [15]. Despite the large sample size and ethnical diversity of the patients included, this does not necessarily resemble the target population in a real-world setting, as most individuals from clinical practices do not meet the eligibility criteria for RCT programmes [17].

Tobacco smoking has been linked with several detrimental effects, including an increased risk of atherosclerosis [18], cardiovascular events [19], nephritis [19], cutaneous manifestations [20], anti-double stranded DNA positivity [21] and reduced efficacy of hydroxychloroquine and belimumab [22–25]. The effect of smoking on different PROs is less understood, with some studies conducted in limited study populations reporting associations between smoking and impaired physical HRQoL [26, 27], increased pain, and fatigue [26, 28]. Particularly, there is a scarcity of data from Scandinavian populations, with previous reports primarily addressing fatigue [28]. Furthermore, whether the negative effects of obesity and smoking on PROs are enhanced by co-exposure to both has not been explored in the SLE population.

In the present study, we investigated associations of obesity and tobacco smoking with an extensive set of PROs in patients with SLE from a Swedish referral centre, comprising HRQoL, pain, fatigue, and functional impairment. Furthermore, we explored whether there was an effect modification between these two factors.

## Methods

### Study design and population

Patients diagnosed with SLE from the Linköping University Hospital meeting the 1982 American College of Rheumatology (ACR) [29] and/or the 2012 Systemic Lupus Erythematosus International Collaborating Clinics Group (SLICC) criteria [30] (*n* = 325) were included in the present cross-sectional analysis of data captured at visits between January 2008 and September 2021. An extensive characterisation of this cohort was recently published [31]. Among consecutive visits, the first visit with complete demographic, clinical, and patient-reported data was selected for the present analysis. Following this approach, we performed complete case analyses, and no missing data imputation was needed.

The study was conducted in compliance with the ethical principles of the Declaration of Helsinki. Written informed consent was obtained from all study participants. Ethical permission for the present investigation was obtained from the Regional Ethics Board in Linköping (ref. M75-08).

### Comparisons with controls from the general population

We further collected information on demographics and self-reported outcome measures from population-based controls. Those individuals were required to be adults residing in areas of Östergötland County or the Stockholm Region, with no diagnosis of a rheumatic disease. Data were collected using electronic forms in the Research Electronic Data Capture (REDCap) system, hosted at Karolinska Institutet, Stockholm, Sweden.

We performed cardinality matching to balance age and sex distributions between cases and controls, while keeping the total number of SLE cases, using the MatchIt package in R, with optimisation performed by GLPK [32]. The resulting subsample of non-SLE controls (*n* = 224) was used for comparisons of the different self-reported measures with the SLE cases, as well as for calculating cut-offs for defining unacceptable outcomes (as detailed below). Supplementary Figure S1 shows the flow diagram for the selection of patients and controls.

### Body mass index and smoking [Exposures]

For patients, body mass index (BMI) was calculated based on anthropometric measurements performed by certified nurses from the Rheumatology Clinic at the Linköping University Hospital. BMI categories were based on the World Health Organization classification: underweight (BMI < 18.5 kg/m^2^), normal weight (18.5 kg/m^2^ ≤ BMI < 25 kg/m^2^), overweight (25 kg/m^2^ ≤ BMI < 30 kg/m^2^), and obesity (BMI ≥ 30 kg/m^2^). For comparisons across BMI groups, we defined normal weight as the reference group.

Smoking status was self-reported and categorised as never, former, and current smoker. For comparisons across smoking status groups, we defined never smoker as the reference group.

### Patient-reported outcomes

PROs evaluated in this analysis included HRQoL, fatigue, pain, overall SLE-related health state, and functional impairment. HRQoL was assessed using the 3-level EQ-5D (EQ-5D-3L) index scores, derived from the United Kingdom value set [33]. Visual analogue scales (VAS; 0–100) were used to capture self-reported fatigue, pain, and overall SLE-related health state within the preceding 7 days. Higher levels represented more severe fatigue and pain, and more impaired overall SLE-related health state. Functional impairment was evaluated using the Swedish version of the Health Assessment Questionnaires Disability Index (HAQ-DI) [34].

Owing to the lack of validated definitions of unacceptable outcomes using VAS scales in the SLE population, we defined unacceptable outcomes as scores equal to or greater than the 90th percentile derived from the matched subsample of non-SLE controls, i.e., scores corresponding to the 10% among non-SLE population-based individuals reporting worst outcomes. Similar procedures have been previously followed for Medical Outcomes Study (MOS) 36-item Short Form (SF-36) data [11, 35]. The calculated cut-offs were: VAS pain score ≥ 35.1; VAS fatigue score ≥ 60.3; VAS overall SLE-related health state score ≥ 50.6.

### Clinical measures

We collected demographic and clinical information for every participant, including age, sex, comorbidities (either directly or using prescribed medications as proxies), disease duration, disease activity, global organ damage, daily glucocorticoid dose and antirheumatic agents prescribed. Disease activity was evaluated using the clinical SLE Disease Activity Index 2000 (cSLEDAI-2 K; serology excluded), whereas organ damage was assessed with the SLICC/ACR Damage Index (SDI) [31].

### Statistical analysis

We compared categorical variables using the chi-squared test, and distributions of continuous variables using the Mann–Whitney *U*-test. We further performed multivariable linear regression analysis to adjust for confounders. For binary outcomes, we estimated crude and adjusted odds ratios (ORs) and 95% confidence intervals (CIs). One set of models was adjusted for age and sex, and a subsequent set of models was further adjusted for disease duration, SDI score, and cSLEDAI-2 K score. The same set of confounders was deemed appropriate for both main exposures. We assessed possible synergistic effects between obesity and smoking (effect modification) on the different binary outcomes by estimating measures of interaction on the multiplicative scale for odds ratio, as well as the relative excess risk due to interaction (RERI) as a measure of additive interaction, using the interactionR package in R [36]. Differences yielding a *P* value < 0.05 were considered statistically significant.

We used the R software version 4.2.1 (R Foundation for Statistical Computing, Vienna, Austria) for data management, statistical analyses, and illustrations.

## Results

### Patient characteristics

Table 1 shows demographics and clinical characteristics of the patients stratified by BMI category, whereas Table 2 shows data stratified by smoking status. Most patients were women of White background. The median age of the cohort was 51 (interquartile range [IQR]: 37–77) years. Furthermore, most patients had adequate disease control, with low median cSLEDAI-2 K scores (0 (IQR: 0–2)) and prednisone equivalent doses (2.5 (IQR: 0.0–5.0) mg/day). Approximately 15% of the individuals were prescribed antidepressants or levothyroxine; no statistically significant differences were observed in the proportions of patients using these medications across BMI or smoking status categories (Supplementary Tables S1–S2).

**Table 1:** Demographic and clinical characteristics of the study population, stratified by BMI category

	Normal weight (*N* = 154)	Underweight (*N* = 5)		Overweight (*N* = 111)		Obese (*N* = 55)	
			*P* value		*P* value		*P* value
Age (years), median (IQR)	44.3 (30.7–59.6)	36.9 (27.8–56.3)	0.615	60.5 (43.6–71.1)	< 0.001	51.7 (41.5–66.6)	0.007
Women, *n* (%)	138 (89.6)	5 (100)	1.000	87 (78.4)	0.012	48 (87.3)	0.634
White, *n* (%)	132 (85.7)	4 (80.0)	0.547	101 (91.0)	0.193	49 (89.1)	0.528
Incident SLE^#^, *n* (%)	56 (36.4)	2 (40.0)	1.000	29 (26.1)	0.078	18 (32.7)	0.628
Disease duration (years), median (IQR)	6.4 (0.8–14.5)	9.1 (2.3–24.0)	0.715	10.7 (2.5–17.3)	0.037	11.2 (0.9–18.9)	0.088
BMI (kg/m^2^), median (IQR)	22.2 (21.1–23.3)	17.8 (15.0–18.2)	< 0.001	27.4 (26.0–28.6)	< 0.001	32.7 (31.0–34.8)	< 0.001
Smoking status, *n* (%)					0.091		0.079
Never	88 (59.9)	1 (20.0)		59 (54.1)		24 (45.3)	
Former	37 (25.2)	4 (80.0)		40 (36.7)		22 (41.5)	
Current	22 (15.0)	0		10 (9.2)		7 (13.2)	
cSLEDAI-2 K, median (IQR)	0.0 (0.0–4.0)	0.0 (0.0–16.0)	0.578	0.0 (0.0–2.0)	0.064	0.0 (0.0–2.0)	0.065
SDI, median (IQR)	1.0 (0.0–2.5)	1.0 (0.0–2.5)	0.498	1.0 (0.0–2.0)	0.146	1.0 (0.0–2.0)	0.035
AMA use, n (%)	113 (73.4)	2 (40.0)	0.130	77 (69.4)	0.475	36 (65.5)	0.265
IS use, *n* (%)	58 (37.7)	4 (80.0)	0.076	40 (36.0)	0.787	22 (40.0)	0.759
Prednisone eq. dose (mg/day), median (IQR)	2.5 (0.0–5.0)	0.0 (0.0–20.0)	0.884	3.8 (0.0–5.3)	0.600	5.0 (0.0–5.0)	0.579

^#^Less than six months from the clinical diagnosis of SLE

*AMA* antimalarial agents, *BMI* body mass index, cSLEDAI-2 K clinical SLE Disease Activity Index 2000, eq. equivalent, IQR interquartile range, *IS* immunosuppressants, SDI SLICC/ACR Damage Index, *SLE* systemic lupus erythematosus

*P* values based on chi-squared tests or Mann–Whitney *U* tests compared with the normal weight group

**Table 2:** Demographic and clinical characteristics of the study population, stratified by smoking status

	Never smoker (*N* = 172)	Former smoker (*N* = 103)		Current smoker (*N* = 39)	
			*P* value		*P* value
Age (years), median (IQR)	47.0 (33.7–63.9)	57.6 (39.9–69.5)	0.006	54.1 (35.0–59.7)	0.940
Women, *n* (%)	150 (87.2)	80 (77.7)	0.038	37 (94.9)	0.174
White, *n* (%)	149 (86.6)	94 (91.3)	0.246	32 (82.1)	0.460
Incident SLE^#^, *n* (%)	62 (36.0)	33 (32.0)	0.499	8 (20.5)	0.063
Disease duration (years), median (IQR)	6.1 (0.6–15.8)	9.1 (0.9–18.4)	0.159	13.4 (4.2–17.0)	0.050
BMI (kg/m^2^), median (IQR)	24.5 (22.3–28.0)	26.3 (22.3–29.3)	0.079	24.6 (21.3–29.8)	0.851
cSLEDAI-2 K, median (IQR)	0.0 (0.0–2.0)	0.0 (0.0–2.0)	0.992	0.0 (0.0–2.0)	0.636
SDI, median (IQR)	0.0 (0.0–1.0)	0.0 (1.0–2.0)	0.026	0.0 (0.0–1.0)	0.875
AMA use, *n* (%)	128 (74.4)	70 (68.0)	0.248	27 (69.2)	0.508
IS use, *n* (%)	59 (34.3)	41 (39.8)	0.358	19 (48.7)	0.092
Prednisone eq. dose (mg/day), median (IQR)	2.5 (0.0–5.0)	4.4 (0.0–7.5)	0.243	5.0 (0.0–7.5)	0.614

^#^Less than six months from the clinical diagnosis of SLE

*AMA* antimalarial agents, *BMI* body mass index, cSLEDAI-2 K clinical SLE Disease Activity Index 2000, eq. equivalent, *IQR* interquartile range, *IS* immunosuppressants, SDI SLICC/ACR Damage Index, *SLE* systemic lupus erythematosus

*P* values based on chi-squared tests or Mann–Whitney *U* tests compared with the never smoker group

### Comparisons with non-SLE controls

After cardinality matching, a subsample of 224 non-SLE controls was kept (Supplementary Figure S1), which was balanced regarding age and sex with the SLE cases. SLE patients reported lower scores than controls from the general population in all self-reported measures (Supplementary Figure S2; Supplementary Table S3). The proportion of SLE patients reporting unacceptable levels of pain was 32.0%, followed by unacceptable levels of fatigue (27.7%), and unacceptable overall SLE-related health state (26.5%).

### Associations between BMI and patient-reported outcomes

In the pooled study population, five patients (1.5%) were classified as underweighted, 111 (34.2%) as overweighted, and 55 (16.9%) as obese (Table 1). Due to the low number of underweighted individuals, statistical comparisons were performed between normal-weight and overweighted or obese individuals.

**Fig. 1:**
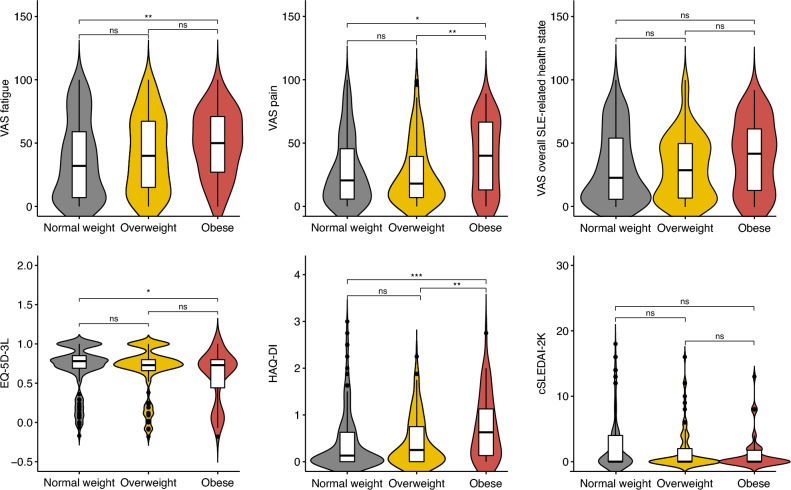
Comparisons of patient-reported outcomes across BMI categories. Violin plots and box plots depicting the score distribution of different patient-reported outcomes and disease activity across BMI categories. Level of significance: ns: *p* > 0.05, *p < 0.05, **p < 0.01, ***p < 0.001. BMI body mass index, cSLEDAI-2 K clinical SLE Disease Activity Index 2000, EQ-5D-3L 3-level EQ-5D questionnaire, *HAQ-DI* Health Assessment Questionnaire Disability Index, *SLE* systemic lupus erythematosus, *VAS* visual analogue scale

Compared with normal-weight individuals, obese SLE patients reported higher VAS fatigue scores (50 (IQR: 27–73) versus 32 (IQR: 7–60); *p* = 0.008), also after adjustments in linear regression models (Fig. 1; Supplementary Table S4). Accordingly, obese patients had 2 times higher odds of reporting unacceptable levels of fatigue (adjusted OR: 2.1; 95% CI 1.0–4.3; *p* = 0.042; Table 3) relative to normal-weight patients. We did not observe differences between overweighted and normal-weight individuals.

**Table 3:** Comparisons of unacceptable patient-reported outcomes across BMI categories

	Normal weight (*N* = 154)	Overweight (*N* = 111)	Obese (*N* = 55)
*Unacceptable levels of fatigue*			
Number of patients (%)	35 (24.1)	33 (30.6)	21 (39.6)
Unadjusted OR (95% CI)	1.0 (Ref.)	1.4 (0.8–2.4) *p* = 0.256	2.1 (1.1–4.0) *p* = 0.034
Adjusted OR (95% CI)	1.0 (Ref.)	1.6 (0.9–3.0) *p* = 0.100	2.5 (1.1–4.5) *p* = 0.020
Fully adjusted OR (95% CI)	1.0 (Ref.)	1.5 (0.8–2.8) *p* = 0.191	2.1 (1.0–4.3) *p* = 0.042
*Unacceptable levels of pain*			
Number of patients (%)	45 (30.4)	30 (27.8)	29 (52.7)
Unadjusted OR (95% CI)	1.0 (Ref.)	0.9 (0.5–1.5) *p* = 0.648	2.6 (1.4–4.8) *p* = 0.004
Adjusted OR (95% CI)	1.0 (Ref.)	1.0 (0.6–1.9) *p* = 0.896	2.8 (1.5–5.6) *p* = 0.002
Fully adjusted OR (95% CI)	1.0 (Ref.)	1.0 (0.5–1.8) *p* = 0.919	3.2 (1.6–6.7) *p* = 0.001
*Unacceptable overall SLE-related health state*			
Number of patients (%)	39 (26.4)	25 (23.1)	21 (38.2)
Unadjusted OR (95% CI)	1.0 (Ref.)	0.8 (0.5–1.5) *p* = 0.559	1.7 (0.9–3.3) *p* = 0.103
Adjusted OR (95% CI)	1.0 (Ref.)	0.9 (0.5–1.7) *p* = 0.800	1.8 (0.9–3.5) *p* = 0.094
Fully adjusted OR (95% CI)	1.0 (Ref.)	0.8 (0.4–1.6) *p* = 0.598	1.9 (0.9–3.8) *p* = 0.087

Proportions of patients reporting unacceptable outcomes within each category, and odds ratios (95% confidence interval) compared with the normal weight group

Adjusted ORs: adjusted for age and sex

Fully adjusted ORs: adjusted for age, sex, disease duration, SDI, clinical SLEDAI-2 K

95% CI 95% confidence interval, BMI body mass index, OR odds ratio, SLE systemic lupus erythematosus

Concerning pain, obese SLE patients reported higher VAS pain scores than both normal-weight and overweighted patients, resulting in higher odds of unacceptable levels of pain compared with the normal-weight group (adjusted OR: 3.2; 95% CI 1.6–6.7; *p* = 0.001).

Furthermore, obese patients reported lower EQ-5D index scores compared with normal-weight patients (0.73 (IQR: 0.44–0.80) versus 0.78 (IQR: 0.69–0.85); *p* = 0.014), which was further confirmed in adjusted linear regression models (β coefficient for BMI:  – 0.165; *p* < 0.001; Supplementary Table S4).

Despite no significant differences in VAS overall SLE-related health state score distributions across groups, increasing BMI was associated with impaired SLE-related health in adjusted linear regression models (β coefficient for BMI: 0.166; *p* = 0.004; Supplementary Table S4). Obesity was associated with two-fold higher odds of severe SLE-related health state compared with normal weight in logistic regression models, but the difference was not statistically significant (Table 3).

Concerning functional impairment, obese individuals reported higher HAQ-DI scores than normal-weight and overweighted individuals, whereas there were no differences between overweighted and normal-weight patients (Fig. 1).

### Associations between smoking and patient-reported outcomes

In the pooled study population, 172 individuals (54.8%) were classified as non-smokers, 103 (32.8%) as former smokers, and 39 (12.4%) as current smokers.

**Fig. 2:**
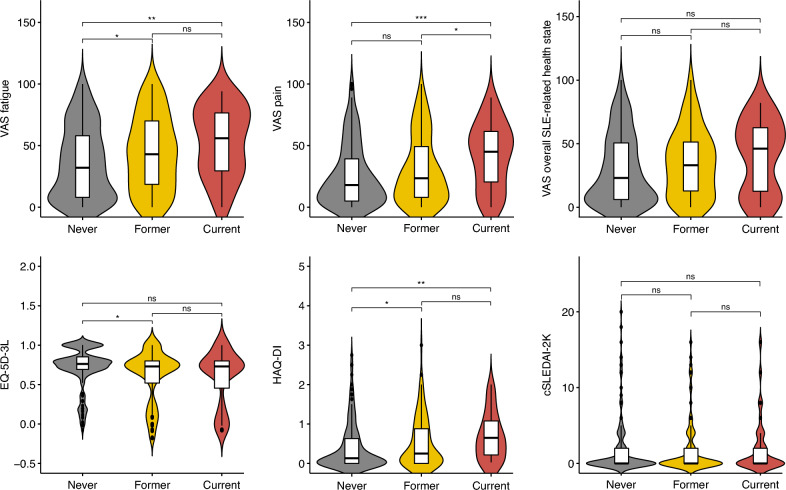
Comparisons of patient-reported outcomes across smoking status categories. Violin plots and box plots depicting the score distribution of different patient-reported outcomes and disease activity across smoking status categories. Level of significance: ns: *p* > 0.05, *p < 0.05, **p < 0.01, ***p < 0.001. cSLEDAI-2 K clinical SLE Disease Activity Index 2000, EQ-5D-3L 3-level EQ-5D questionnaire, *HAQ-DI* Health Assessment Questionnaire Disability Index, *SLE* systemic lupus erythematosus, *VAS* visual analogue scale

When compared with non-smokers, both former and current smokers reported higher VAS fatigue scores (Fig. 2; Supplementary Table S5). In adjusted regression analysis, current smokers had nearly 3 times higher odds of severe fatigue than non-smokers (adjusted OR: 2.8; 95% CI 1.3–5.9; *p* = 0.006; Table 4).

**Table 4:** Comparisons of unacceptable patient-reported outcomes across smoking status categories

	Never smoker (*N* = 172)	Former smoker (*N* = 103)	Current smoker (*N* = 39)
*Unacceptable levels of fatigue*			
Number of patients (%)	41 (24.0)	30 (30.3)	18 (46.2)
Unadjusted OR (95% CI)	1.0 (Ref.)	1.4 (0.8–2.4) *p* = 0.256	2.7 (1.3–5.6) *p* = 0.007
Adjusted OR (95% CI)	1.0 (Ref.)	1.5 (0.8–2.7) *p* = 0.160	2.6 (1.3–5.4) *p* = 0.009
Fully adjusted OR (95% CI)	1.0 (Ref.)	1.5 (0.8–2.8) *p* = 0.152	2.8 (1.3–5.9) *p* = 0.006
*Unacceptable levels of pain*			
Number of patients (%)	48 (27.9)	32 (32.0)	23 (59.0)
Unadjusted OR (95% CI)	1.0 (Ref.)	1.2 (0.7–2.1) *p* = 0.475	3.7 (1.8–7.7) *p* < 0.001
Adjusted OR (95% CI)	1.0 (Ref.)	1.3 (0.8–2.3) *p* = 0.306	3.5 (1.7–7.4) *p* < 0.001
Fully adjusted OR (95% CI)	1.0 (Ref.)	1.4 (0.8–2.6) *p* = 0.220	3.8 (1.8–8.2) *p* < 0.001
*Unacceptable overall SLE-related health state*			
Number of patients (%)	43 (25.0)	26 (26.0)	16 (41.0)
Unadjusted OR (95% CI)	1.0 (Ref.)	1.1 (0.6–1.8) *p* = 0.855	2.1 (1.0–4.3) *p* = 0.047
Adjusted OR (95% CI)	1.0 (Ref.)	1.1 (0.6–2.0) *p* = 0.673	1.9 (0.9–4.0) *p* = 0.074
Fully adjusted OR (95% CI)	1.0 (Ref.)	1.1 (0.6–2.0) *p* = 0.808	2.0 (0.9–4.3) *p* = 0.067

Proportions of patients reporting unacceptable outcomes within each category, and odds ratios (95% confidence interval) compared with the never smoker group

Adjusted ORs: adjusted for age and sex

Fully adjusted ORs: adjusted for age, sex, disease duration, SDI, clinical SLEDAI-2 K

95% *CI 95*% confidence interval, *OR* odds ratio, *SLE* systemic lupus erythematosus

The current smokers reported higher VAS pain scores than both never smokers and former smokers, resulting in nearly 4 times higher odds of unacceptable levels of pain compared with non-smokers in adjusted models (adjusted OR: 3.8; 95% CI 1.8–8.2; *p* < 0.001).

Concerning HRQoL, former, but not current, smokers reported slightly lower EQ-5D scores compared with non-smokers (0.73 (IQR: 0.52–0.80) versus 0.76 (IQR: 0.69–0.85); *p* = 0.014).

The mean VAS overall SLE-related health state scores and the proportion of patients experiencing an unacceptable state were not significantly different across groups.

Finally, we observed a dose − response relationship between smoking status and functional impairment, with former (0.25 (IQR: 0.0–0.88); *p* = 0.031) and current smokers (IQR: 0.63 (0.19–1.07); *p* = 0.001) reporting higher HAQ-DI scores than non-smokers (0.13 (IQR: 0.00–0.63)).

### Effect modification between BMI and smoking

We next assessed whether the effect of BMI on the different PROs differed according to smoking status, by estimating measures of additive and multiplicative interaction (Supplementary Tables S6–S8). There were no indications of a positive interaction between obesity and smoking status for unacceptable fatigue, unacceptable levels or pain, or unacceptable experience of overall SLE-related health state. Importantly, compared with non-obese and non-smoker individuals, non-obese and smoker, smoker and non-obese, as well as obese and smoker patients presented higher odds of unacceptable levels of pain and fatigue.

## Discussion

In a population of SLE patients from the Östergötland County in Sweden, we studied associations between (i) BMI and (ii) tobacco smoking and several PROs, and explored whether there was a synergistic effect between these two exposures. Patients with SLE experienced considerably poorer HRQoL and greater levels of fatigue, pain, and functional impairment compared with population-based non-SLE individuals matched for age, sex, and region of residence. Although disease activity levels did not differ significantly between obese and normal weight patients, obese SLE patients reported lower HRQoL, and higher levels of fatigue, pain, and functional impairment than normal weight individuals. Similarly, the current smokers reported higher levels of fatigue, pain, and functional impairment than never smokers. The observed associations were independent of age, sex, disease duration, disease activity, and organ damage. However, there was no evidence of a synergistic effect between increased BMI and smoking on any of the self-reported outcomes.

Our study adds to a growing body of evidence linking increased BMI with unsatisfactory self-reports in several domains. Consistent with our previous report from a large population from an RCT setting [15], pain, fatigue, and functional impairment were herein amongst the most affected domains in obese compared with normal weight SLE patients. While validation of the cut-offs to signify unacceptable patient experience in SLE populations is needed, our observations collectively underscore the clinical significance of the reported associations, evidenced by the absolute differences > 10 mm in mean VAS pain and fatigue scores between normal weight and obese patients, along with HAQ-DI scores showing differences > 0.22 [37, 38]; these differences exceeded half a standard deviation for each measure [39]. Furthermore, the observations of increasingly unfavourable outcomes with higher BMI, along with previous associations between excess adiposity and worse PROs [16], give lend to a biological plausibility for these associations, and advocate for weight control strategies as potential interventions for improving PROs. Nevertheless, multiple interventions are available for weight management (e.g., diet, physical exercise, pharmacotherapy, surgery, and combinations thereof), each expected to exhibit varying degree of efficacy in improving diverse health-related outcomes [40]. This variability arises, partly, because the effects of some interventions may not be solely mediated through changes in body weight. Consequently, RCTs are essential to estimate the effects of different weight management strategies on various PROs in SLE populations that are at risk of experiencing poor outcomes.

When compared with patients who had not been exposed to tobacco, smokers reported reduced HRQoL and higher levels of fatigue, pain, and functional impairment. The differences were notably more pronounced in current smokers than in those who had quit smoking, resulting in a substantial absolute risk (40–60%) of unacceptable outcomes. Even though we lacked detailed information on smoking, these findings are consistent with previous reports linking pack-years of smoking and poor physical HRQoL [27], further supporting the clinical significance of these associations. Mechanisms through which smoking could increase pain and fatigue include reduced lung and cardiovascular function, skeletal muscle dysfunction, and low-grade systemic inflammation [41, 42]. Furthermore, some evidence supports the hypothesis that smoking contributes to immune dysregulation in SLE, including associations between smoking and (i) occurrence of anti-dsDNA antibodies [21], (ii) increased BAFF [43], and (iii) a reduced effect to hydroxychloroquine [22] and belimumab in SLE [23–25], as well as methotrexate in rheumatoid arthritis [44, 45]; in the case of biologics, this could presumably be attributed to the generation of anti-drug antibodies [46]. To get a broader and deeper understanding of the pathways involved, gene polymorphism-smoking interaction analyses constitute an appealing approach, as previously attempted for other outcomes [19, 47, 48].

Interestingly, the detrimental associations between obesity or smoking and different PROs remained even in patients who had an adequate disease control. It is worth nothing that such associations with increasing BMI have also been reported after a one-year intervention in an RCT setting [49, 50], collectively suggesting that obese patients and smokers are at risk of experiencing adverse outcomes regardless of the underlying degree of clinical activity. As per the recently released EULAR recommendations for the non-pharmacological management of SLE and systemic sclerosis, physical exercise and smoking cessation should be regarded as fundamental components of patient care [12]. During a shared decision-making process, we expect that patients may be more motivated to adhere to such interventions, driven by evidence-based information that some of the outcomes that are most relevant for them – such as pain, fatigue, and daily functioning – may improve. We advocate for systematically measuring PROs with validated instruments during clinical visits as a crucial step towards identification of needs and implementation of strategies to address those.

In the interaction analyses, we did not observe a synergistic effect between obesity and smoking on any of the binary outcomes explored. Instead, we could corroborate that even carrying one of the risk factors (being obese and non-smoker, or of normal weight and smoker) was associated with an increased probability of experiencing unacceptable outcomes, particularly pain and fatigue. Overall, these observations provide support for interventions addressing smoking and obesity as a strategy for improving these outcomes where pharmacological agents apparently have limited effects [51, 52]. Although the current evidence on the efficacy of non-pharmacological interventions in patients with SLE mostly encompasses unimodal interventions [40], weight control and smoking cessation strategies could be delivered alone or in combination, tailored to the patients’ needs and preferences [12]. Importantly, the optimal target population, setting, delivery method, feasibility, and efficacy of more complex multimodal interventions are heavily underexplored.

Our study has certain limitations. The cross-sectional design prevented us from establishing causality. Furthermore, the number of individuals included was relatively low, hindering statistical power to detect some effects and interactions. Smoking status was self-reported by the patients, and we lacked information on pack-years, creating a risk of misclassification in our analysis. Moreover, we lacked information on heredity and some relevant comorbidities; we mitigated the latter by including comparisons of the use of certain medications, which we used as proxies for comorbid conditions. Finally, even though we adjusted for some demographic and disease-specific covariates, we cannot exclude residual confounding due to socioeconomical variables, such as income and marital status [21, 28, 53]. The study population consisted predominantly of women of White background who received care in an academic centre in mid-Sweden. Given the known impact of social determinants on obesity, tobacco smoking, and disease outcomes, including self-perception of health [54], our findings cannot be considered directly generalisable to all SLE populations.

The strengths of our study include the systematic capture of multiple PROs in routine clinical practice – encompassing HRQoL, fatigue, pain, overall SLE-related health state, and functional impairment –, which, to our knowledge, constitutes the most comprehensive description of PROs in relation to obesity and smoking in a Scandinavian SLE population. A major strength was the high degree of data completeness, which allowed us to perform multivariable regression analyses, despite the relatively limited sample size. The very high coverage of SLE patients from the county of Östergötland in the KLURING cohort, which has been estimated to be ≥ 97% of all cases [31], eliminated the selection bias in our investigation. Furthermore, the inclusion of controls and the use of matching procedures allowed us to perform comparisons between the SLE population and non-SLE population-based individuals and derive cut-offs for unacceptable outcomes specific for our SLE population.

In conclusion, we herein report from a single-centre Swedish academic setting that obese SLE patients experienced lower HRQoL and higher levels of fatigue, pain, and functional impairment than normal weight individuals, and that current smokers experienced higher levels of fatigue, pain, and functional impairment than never smokers. Interventions targeting obesity and smoking, tailored to meet the individual patient’s needs and preferences, may not only improve metabolic and cardiovascular outcomes, but also pain, fatigue, and daily function, disease components which are of particular interest for the patients.

### Supplementary Information

Below is the link to the electronic supplementary material.

Supplementary file1 (PDF 680 KB)

Supplementary file2 (DOCX 980 KB)

## Data Availability

Data supporting the findings described in this manuscript are available upon reasonable request.

## Declarations

### Congress abstract publication

Data from the current work have been previously presented at one scientific meeting [55].
