# Supplementary material for: Obesity and tobacco smoking are independently associated with poor patient-reported outcomes in SLE: a cross-sectional study
Source: Rheumatol Int. 2024 Mar 7;44(5):851–61. doi: 10.1007/s00296-024-05546-z (PMC10980611; doi:10.1007/s00296-024-05546-z)
Supplement: Supplementary file 1 — Supplementary file1 (PDF 680 KB) [file 296_2024_5546_MOESM1_ESM.pdf]

**Supplementary Table S1. Comorbidities of the study population, stratified by BMI category.**

| | Normal weight (N = 154) | Underweight (N = 5) | | Overweight (N = 111) | | Obese (N = 55) | |
|---|---|---|---|---|---|---|---|
| | | | P value | | P value | | P value |
| Raynaud's phenomenon, n (%) | 51 (33.1) | 3 (60.0) | 0.233 | 23 (20.7) | 0.028 | 8 (14.5) | 0.011 |
| Secondary Sjögren's syndrome, n (%) | 31 (20.1) | 0 | 0.983 | 26 (23.4) | 0.520 | 15 (27.3) | 0.274 |
| Use of levothyroxine (ever), n (%) | 18 (11.7) | 0 | 0.989 | 21 (18.9) | 0.104 | 9 (16.4) | 0.377 |
| Use of antidepressants (ever), n (%) | 22 (14.3) | 0 | 0.989 | 16 (14.4) | 0.976 | 12 (21.8) | 0.197 |
| Use of antihypertensive drugs (ever), n (%) | 56 (36.4) | 3 (60.0) | 0.298 | 60 (54.1) | 0.004 | 38 (69.1) | <0.001 |
| Use of lipid-lowering drugs (ever), n (%) | 21 (13.6) | 0 | 0.989 | 18 (16.2) | 0.559 | 17 (30.9) | 0.005 |

P values derived from univariable logistic regression models with the normal weight group as reference.

**Supplementary Table S2. Comorbidities of the study population, stratified by smoking status.**

| | Never smoker (N = 172) | Former smoker (N = 103) | | Current smoker (N = 39) | |
|---|---|---|---|---|---|
| | | | P value | | P value |
| Raynaud's phenomenon, n (%) | 42 (24.4) | 26 (25.2) | 0.878 | 15 (38.5) | 0.078 |
| Secondary Sjögren's syndrome, n (%) | 35 (20.3) | 24 (23.3) | 0.564 | 10 (25.6) | 0.467 |
| Use of levothyroxine (ever), n (%) | 27 (15.7) | 12 (11.7) | 0.353 | 7 (17.9) | 0.730 |
| Use of antidepressants (ever), n (%) | 21 (12.2) | 20 (19.4) | 0.107 | 8 (20.5) | 0.179 |
| Use of antihypertensive drugs (ever), n (%) | 71 (41.3) | 61 (59.2) | 0.004 | 19 (48.7) | 0.397 |
| Use of lipid-lowering drugs (ever), n (%) | 24 (14.0) | 22 (21.4) | 0.114 | 7 (17.9) | 0.526 |

P values derived from univariable logistic regression models with the never smoker group as reference.

**Supplementary Table S3.** Comparisons of patient-reported outcomes between SLE cases and population-based non-SLE matched controls.

| | **SLE cases (N= 325)** | **Matched controls (N= 224)** | **P value** |
|---|---|---|---|
| Age (years), median (IQR) | 50.1 (36.6–66.6) | 45.0 (37.0–60.0) | 0.053 |
| Women, n (%) | 278 (85.5) | 181 (80.8) | 0.175 |
| VAS fatigue (mm), median (IQR) | 38.0 (13.0–67.5) | 20.0 (10.0–41.5) | <0.001 |
| VAS pain (mm), median (IQR) | 22.0 (7.0–48.3) | 7.00 (0–19.3) | <0.001 |
| VAS overall health state/SLE-related health state (mm), median (IQR) | 29.0 (7.0–53.0) | 10.0 (2.8–23.0) | <0.001 |
| HAQ-DI, median (IQR) | 0.25 (0–0.75) | 0 (0–0) | <0.001 |
| EQ-5D-3L index score, median (IQR) | 0.73 (0.64–0.80) | 1.00 (0.80–1.00) | <0.001 |
| EQ-5D-3L full health state, n (%) | 62 (19.1) | 111 (49.6) | <0.001 |

EQ-5D-3L: 3-level EQ-5D; HAQ-DI: Health Assessment Questionnaire Disability Index; IQR: interquartile range; SLE: systemic lupus erythematosus; VAS: visual analogue scale.

**Supplementary Table S4**. Linear regression models for the associations between BMI and different patient-reported outcomes, disease activity, and organ damage.

| | Unstandardised coefficient | Standardised coefficient | Standard error | P value |
|---|---|---|---|---|
| **Unadjusted models** | | | | |
| VAS fatigue | 0.947 | 0.151 | 0.352 | 0.007 |
| VAS pain | 0.996 | 0.180 | 0.307 | 0.001 |
| VAS overall SLE-related health state | 0.714 | 0.125 | 0.320 | 0.026 |
| EQ-5D-3L | -0.008 | -0.130 | 0.004 | 0.019 |
| HAQ-DI | 0.021 | 0.163 | 0.007 | 0.003 |
| cSLEDAI-2K | -0.138 | -0.178 | 0.043 | 0.001 |
| SDI | 0.025 | 0.062 | 0.023 | 0.278 |
| **Adjusted models** | | | | |
| VAS fatigue | 1.101 | 0.176 | 0.372 | 0.003 |
| VAS pain | 1.171 | 0.214 | 0.319 | <0.001 |
| VAS overall SLE-related health state | 0.945 | 0.166 | 0.322 | 0.004 |
| EQ-5D-3L | -0.011 | -0.165 | 0.113 | <0.001 |
| HAQ-DI | 0.022 | 0.169 | 0.007 | 0.002 |
| cSLEDAI-2K | -0.106 | -0.136 | 0.044 | 0.017 |
| SDI | -0.029 | -0.071 | 0.020 | 0.154 |

Adjusted models: adjusted for age, sex, disease duration, SDI, cSLEDAI-2K.

BMI: body mass index; cSLEDAI-2K: clinical SLE Disease Activity Index 2000; EQ-5D-3L: 3-level EQ-5D; HAQ-DI: Health Assessment Questionnaire Disability Index; SDI: SLICC/ACR Damage Index; SLE: systemic lupus erythematosus; VAS: visual analogue scale.

**Supplementary Table S5**. Linear regression models for the associations between smoking status and different patient-reported outcomes, disease activity, and organ damage.

| | Smoking status* | Unstandardised coefficient | Standardised coefficient | Standard error | P value |
|---|---|---|---|---|---|
| **Unadjusted models** | | | | | |
| VAS fatigue | F | 7.73 | 0.12 | 3.70 | 0.037 |
| | C | 17.11 | 0.19 | 5.19 | 0.001 |
| VAS pain | F | 4.55 | 0.08 | 3.23 | 0.161 |
| | C | 16.10 | 0.20 | 4.56 | <0.001 |
| VAS overall SLE-related health state | F | 5.24 | 0.09 | 3.39 | 0.124 |
| | C | 9.94 | 0.12 | 4.78 | 0.038 |
| EQ-5D-3L | F | -0.096 | -0.151 | 0.037 | 0.019 |
| | C | -0.112 | -0.125 | 0.052 | 0.009 |
| HAQ-DI | F | 0.13 | 0.10 | 0.08 | 0.081 |
| | C | 0.28 | 0.15 | 0.11 | 0.010 |
| cSLEDAI-2K | F | -0.04 | 0.00 | 0.46 | 0.938 |
| | C | 0.10 | 0.01 | 0.66 | 0.879 |
| SDI | F | 0.60 | 0.16 | 0.23 | 0.009 |
| | C | 0.01 | 0.00 | 0.32 | 0.982 |
| **Adjusted models** | | | | | |
| VAS fatigue | F | 9.39 | 0.15 | 3.81 | 0.014 |
| | C | 17.21 | 0.20 | 5.19 | 0.001 |
| VAS pain | F | 5.42 | 0.10 | 3.32 | 0.103 |
| | C | 15.84 | 0.20 | 4.53 | <0.001 |
| VAS overall SLE-related health state | F | 5.79 | 0.10 | 3.36 | 0.086 |
| | C | 9.50 | 0.12 | 4.60 | 0.040 |
| EQ-5D-3L | F | -0.078 | -0.12 | 0.037 | 0.038 |
| | C | -0.110 | -0.12 | 0.051 | 0.031 |
| HAQ-DI | F | 0.06 | 0.05 | 0.07 | 0.416 |
| | C | 0.27 | 0.15 | 0.10 | 0.006 |
| cSLEDAI-2K | F | 0.15 | 0.02 | 0.47 | 0.751 |
| | C | 0.34 | 0.03 | 0.65 | 0.596 |
| SDI | F | 0.33 | 0.08 | 0.20 | 0.097 |
| | C | -0.05 | -0.01 | 0.27 | 0.849 |

Adjusted models: age, sex, disease duration, SDI, cSLEDAI-2K.

cSLEDAI-2K: clinical SLE Disease Activity Index 2000; EQ-5D-3L: 3-level EQ-5D; HAQ-DI: Health Assessment Questionnaire Disability Index; SDI: SLICC/ACR Damage Index; SLE: systemic lupus erythematosus; VAS: visual analogue scale.

* C: current smoker; F: former smoker. Reference: never smoker.

**Supplementary Table S6**. Interaction between obesity and smoking status for unacceptable levels of fatigue.

| | Non-smoker | Ever smoker | Effect of smoking ever within the strata of obese |
|---|---|---|---|
| **Non-obese** | 1.0 (reference) | 2.1 (1.2 to 3.7) p=0.010 | 2.1 (1.2 to 3.7) p=0.010 |
| **Obese** | 2.8 (1.1 to 6.8) p=0.028 | 2.4 (1.0 to 5.5) p=0.047 | 0.9 (0.3 to 2.6) p=0.794 |
| **Effect of obese within the strata of smoking ever** | 2.8 (1.1 to 6.8) p=0.028 | 1.1 (0.5 to 2.6) p=0.801 | |
| Effect modification on multiplicative scale (95% CI): 0.4 (0.1 to 1.4) p=0.156 | | | |
| Effect modification on additive scale (RERI; 95% CI): -1.5 (-4.7 to 1.6) p=0.826 | | | |

Data are presented as individual and joint odds ratios (95% confidence interval), with non-smoker and non-obese as the reference. Measures of additive and multiplicative effect modification are provided.

RERI: Relative Excess Risk due to Interaction.

**Supplementary Table S7**. Interaction between obesity and smoking status for unacceptable levels of pain.

| | **Non-smoker** | **Ever smoker** | **Effect of smoking ever within the strata of obese** |
|---|---|---|---|
| **Non-obese** | 1.0 (reference) | 2.4 (1.4 to 4.1) p=0.002 | 2.4 (1.4 to 4.1) p=0.002 |
| **Obese** | 7.2 (2.8 to 18.3) p<0.001 | 2.9 (1.3 to 6.7) p=0.011 | 0.4 (0.1 to 1.3) p=0.115 |
| **Effect of obese within the strata of smoking ever** | 7.2 (2.8 to 18.3) p<0.001 | 1.2 (0.5 to 2.8) p=0.626 | |
| Effect modification on multiplicative scale (95% CI): 0.2 (0.1 to 0.6) p=0.006 | | | |
| Effect modification on additive scale (RERI; 95% CI): -5.6 (-17.1 to 0.4) p=0.942 | | | |

Data are presented as individual and joint odds ratios (95% confidence interval), with non-smoker and non-obese as the reference. Measures of additive and multiplicative effect modification are provided.

RERI: Relative Excess Risk due to Interaction.

**Supplementary Table S8**. Interaction between obesity and smoking status for unacceptable overall SLE-related health state.

| | **Non-smoker** | **Ever smoker** | **Effect of smoking ever within the strata of obese** |
|---|---|---|---|
| **Non-obese** | 1.0 (reference) | 1.6 (0.9 to 2.9) p=0.100 | 1.6 (0.9 to 2.9) p=0.100 |
| **Obese** | 3.2 (1.3 to 7.8) p=0.012 | 2.0 (0.8 to 4.7) p=0.123 | 0.6 (0.2 to 1.9) p=0.409 |
| **Effect of obese within the strata of smoking ever** | 3.2 (1.3 to 7.8) p=0.012 | 1.2 (0.5 to 2.9) p=0.671 | |
| Effect modification on multiplicative scale (95% CI): 0.4 (0.1 to 1.3) p=0.133 | | | |
| Effect modification on additive scale (RERI; 95% CI): -1.8 (-5.0 to 1.4) p=0.864 | | | |

Data are presented as individual and joint odds ratios (95% confidence interval), with non-smoker and non-obese as the reference. Measures of additive and multiplicative effect modification are provided.

RERI: Relative Excess Risk due to Interaction.
