# Supplementary material for: Obesity and tobacco smoking are independently associated with poor patient-reported outcomes in SLE: a cross-sectional study
Source: Rheumatol Int. 2024 Mar 7;44(5):851–61. doi: 10.1007/s00296-024-05546-z (PMC10980611; doi:10.1007/s00296-024-05546-z)
Supplement: Supplementary file 2 — Supplementary file2 (DOCX 980 KB) [file 296_2024_5546_MOESM2_ESM.docx]

**Supplementary Figure S1. Flow diagram of participant disposition.** BMI body mass index, PROs patient-reported outcomes, SLE systemic lupus erythematosus.

**Supplementary Figure S2. Comparisons of patient-reported outcomes between SLE cases and population-based non-SLE matched controls.**

Violin plots and box plots depicting the score distribution of different patient-reported outcomes and disease activity across smoking status categories.

Level of significance: ns: p>0.05, *: p<0.05, **: p<0.01, ***: p<0.001.

EQ-5D-3L 3-level EQ-5D questionnaire, HAQ-DI Health Assessment Questionnaire Disability Index, SLE systemic lupus erythematosus, VAS visual analogue scale.
